# COVID-19 Seroprevalence in Canada Modelling Waning and Boosting COVID-19 Immunity in Canada a Canadian Immunization Research Network Study

**DOI:** 10.3390/vaccines10010017

**Published:** 2021-12-23

**Authors:** David W. Dick, Lauren Childs, Zhilan Feng, Jing Li, Gergely Röst, David L. Buckeridge, Nick H. Ogden, Jane M. Heffernan

**Affiliations:** 1 Mathematics and Statistics, Centre for Disease Modelling, York University, Toronto, ON M3J 1P3, Canada; dwdick@yorku.ca; 2 Department of Mathematics, Virginia Tech, Blacksburg, VA 24061, USA; lchilds@vt.edu; 3 Department of Mathematics, Purdue University, West Lafayette, IN 46202, USA; zfeng@math.purdue.edu; 4 National Science Foundation, Alexandria, VA 22314, USA; 5 Department of Mathematics, California State University, Northridge, CA 91330, USA; jing.li@csun.edu; 6 Department of Mathematics, University of Szeged, 6720 Szeged, Hungary; rost@math.u-szeged.hu; 7 Epidemiology and Biostatistics, School of Population and Global Health, McGill University, Montreal, QC H3A 0G4, Canada; david.buckeridge@mcgill.ca; 8 National Microbiology Laboratory, Public Health Risk Sciences Division, Public Health Agency of Canada, St. Hyacinthe, QC J2S 2M2, Canada; nicholas.ogden@canada.ca

**Keywords:** seroprevalence, COVID-19, infectious disease modelling, mathematical epidmiology

## Abstract

COVID-19 seroprevalence changes over time, with infection, vaccination, and waning immunity. Seroprevalence estimates are needed to determine when increased COVID-19 vaccination coverage is needed, and when booster doses should be considered, to reduce the spread and disease severity of COVID-19 infection. We use an age-structured model including infection, vaccination and waning immunity to estimate the distribution of immunity to COVID-19 in the Canadian population. This is the first mathematical model to do so. We estimate that 60–80% of the Canadian population has some immunity to COVID-19 by late Summer 2021, depending on specific characteristics of the vaccine and the waning rate of immunity. Models results indicate that increased vaccination uptake in age groups 12–29, and booster doses in age group 50+ are needed to reduce the severity COVID-19 Fall 2021 resurgence.

## 1. Introduction

The COVID-19 pandemic continues to affect the lives of Canadians. Despite increasing vaccination uptake, of first and second doses, and great decreases in COVID-19 cases of late, questions remain as to the future of the COVID-19 pandemic in this country, and any future need for COVID-19 vaccines to tackle COVID-19 resurgence.

A required step in understanding possibilities of resurgence or future vaccine needs lies in the determination and quantification of immunity in the Canadian population. Seroprevalence studies in different population cohorts have been conducted (see [1,2] for examples, and [3] for more information, many others are underway—see [4] for details) which can inform immunity distribution calculations. A recent statistical study by the COVID-19 Immunity Task Force (CITF), which incorporates different population seroprevalence studies into their analysis, estimated that the Canadian population has some immunity against COVID-19: due to infection, 5.4% (95% CrI: 0.6 to 15.8) as of 31 May 2021; due to infection and vaccination, 44.9% (95% CrI: 44.2 to 45.8) as of same date [5] (Credible interval (CrI)). The CITF [5] is the only working group that we know of that has attempted to quantify seroprevalence in the Canadian population.

It is of interest to provide other estimates of COVID-19 immunity in the Canadian population. Mathematical models of COVID-19 infection and vaccination can be used to estimate immunity distributions. In a previous study, we developed a mathematical model of COVID-19 infection and vaccination [6]. The model tracks infection and immunity status by age. An important difference of our model over other models of COVID-19 is that it incorporates differential outcomes of immunity, determined by infection severity, which is ultimately related to the prevalence of comorbidities in the Canadian population by age. A second important difference is that our model includes the effects of waning immunity, whereby immunity protection gained from infection or vaccination can decay over time. The model therefore has the capacity to provide time varying estimates of COVID-19 seroprevalence, which in turn can be used to inform public health decision-makers on vaccination policy i.e., targeting primary coverage to specific age groups, or booster doses.

In the current study we use our mathematical model to determine distributions of immunity in the Canadian population, by age, from infection, and from vaccination. The model is fit to daily COVID-19 incidence data up to 27 June 2021 [7], and incorporates actual (up to 27 June 2021) and projected (to September 2021) coverage of the first and second doses of COVID-19 vaccines [7,8]. We then use the model to quantify distributions of immunity from January 2020 to March 2022, given different assumed characteristics of the vaccines against various variants of concern (i.e., protection from infection, protection from severe disease), and different rates of waning immunity. In summary, we find that 60–80% of the Canadian population has some immunity to COVID-19 by late Summer 2021, depending on specific characteristics of the vaccine and the waning rate of immunity. Model results also indicate that increased vaccination uptake in age groups 12–29, and booster doses in age group 50+ are needed to reduce the severity COVID-19 Fall 2021 resurgence.

## 2. Methods

**Figure 1:**
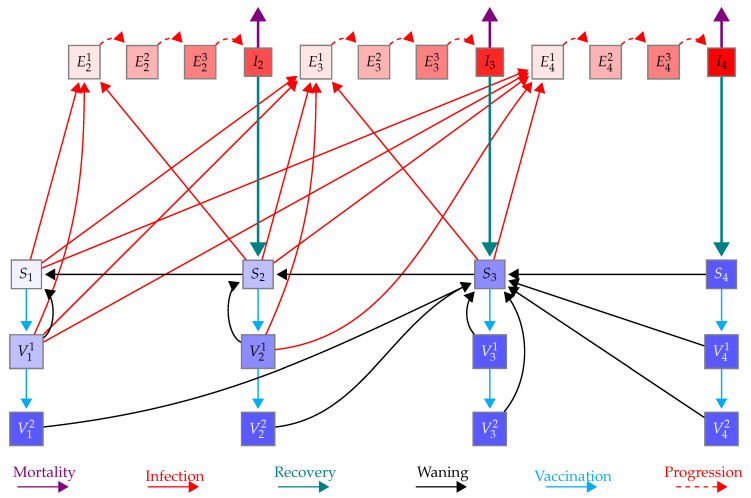
Schematic of the model for one age group. Here, $S_{1}$, $S_{2}$, $S_{3}$, and $S_{4}$ (purple shaded boxes) represent susceptible individuals who are immunologically naive, have some, moderate, and full immunity, respectively. $I_{2}$, $I_{3}$, and $I_{4}$ (red boxes) represent infected individuals with mild, moderate and severe symptoms, respectively, who will develop some, moderate, and full immunity once recovered (teal solid line), respectively. $V_{i}^{j}$(i=1,2,3,4,j=1,2) represent vaccinated individuals from the $S_{i}$ classes (i=1,2,3,4) after j=1,2 doses of vaccine given a two-dose schedule. $E_{i}^{k}$(i=2,3,4;k=1,2,3) represent exposed individuals (infected, asymptomatic, not infectious) with progressive stages k=2,3,4 that will experience mild $I_{2}$, moderate $I_{3}$, and severe $I_{4}$ symptoms. Susceptible and vaccinated individuals can be infected and move to the exposed classes (red lines). Susceptible and vaccinated classes at the same location on the immunity continuum have similar characteristics. Immunity gained from infection and vaccination can wane (black lines). The bottom of the figure lists all the legends.

We implemented a model of COVID-19 infection with age structure (i.e., groups 0–4, 5–9, ⋯, 75+ years). A flow diagram of the model is shown in Figure 1 for one age group. The model is based on a Susceptible-Exposed-Infected-Vaccinated-Susceptible model structure (SEIVS). We use $S_{i}$, $E_{j}^{k}$, $I_{j}$, and $V_{i}^{\ell }$ to denote the number of susceptible, exposed, infected and vaccinated individuals in each age group, where *i* (1≤i≤4) denotes immune status, *j* (2≤j≤4) denotes symptom severity, *k* (1≤k≤3) represents stages in the exposed class (to obtain Gamma-distributed exposed sojourns), and ℓ=1,2 denotes the number of doses of vaccine that individuals have received.

A detailed description of the mathematical model can be found in [6] and in the Appendix. Briefly, we assume that mild ($I_{2}$), moderate ($I_{3}$), or severe ($I_{4}$) disease can be experienced upon infection, and that the probability of mild, moderate, or severe disease is determined by the comorbidity status in each age group [9]. We assume that all $I_{4}$ infecteds will be reported. We also assume that some fraction of $I_{3}$ will get tested and will be reported. Finally, we account for a small number of reported cases in $I_{3}$ and $I_{2}$ that will be tested because of contact tracing.

We assume that immunity gained after infection correlates with the severity of infection, such that higher levels of immunity are gained in individuals that have experienced more severe disease [10,11,12]. Immunity gained from vaccination is also implemented in the model, using actual Canadian vaccination roll-out data and projections [7,8]. Three different types of vaccine characteristics are considered which reflect protective capacities against infection and/or disease (given different variants of concern (VOC)) of the vaccines used in the Canadian population [13,14,15,16,17,18,19]. Characteristics of the vaccines are listed in Table 1.

In addition to modelling the gain of immunity, we also consider immunity decay in the population. Different waning rates are considered such that immunity wanes on average 1 year or 3 years between *S* and *V* classes, and consecutive *S* classes, which gives waning from full immunity to full susceptibility over 3 years or 9 years, respectively. For comparison, we also consider the case when immunity does not wane.

Finally, public health mitigation is incorporated into the model using modified contact matrices for home, school, work, and other types of contacts between age groups. Figure A2 plots the mitigation windows (gray bars) and the percent reduction in contacts gained from the contact matrix mitigation modifications in each window (x’s). The model also collectively accounts for compliance to social distancing and mask wearing, changes in testing and contact tracing rates, and changes in transmission due to VOCs and weather. This is done using a parameter κ that is determined from a model fit to daily incidence data.

Using our mathematical model, we track the distribution of immunity in the Canadian population over time, given different characteristics of protective capacity from the vaccine, and different assumptions with respect to the waning rate of immunity. There are nine scenarios that we consider altogether. We fit the model for each scenario from 25 January 2020 to 27 June 2021.

Projecting forward from the model fit, we modify the contact matrices corresponding to phase 1 in June to represent easing of lockdown restrictions across Canada with the schools remaining closed. In July, we increase contacts in public spaces, to reflect different reopening steps taken in Canadian jurisdictions. In September the contact restrictions are eased to phase 2, representing school reopening and easing of restrictions at work. For a detailed description of the mitigation phases see Table A3 and Table A4.

Behaviour is likely to relax over the summer and into the fall, increasing transmission. In July 2021 many jurisdictions relaxed social distancing rules. To reflect this, and the prevalence of the delta VOC, we increase κ by a factor of 2. Reductions in transmissibility due to weather should only affect the summer months. We suggest, therefore, that κ can be increased in September 2021. We compare model results with no change in κ in September 2021, a 20% increase to reflect increased transmissibility due to changes in the weather [20], and a 38% increase (to allow for consideration of introduction of a new VOC). Figure A2 provides an example of the contact rate change assuming an increase by a factor of 2 in the summer months following by a 20% increase to account for changes in weather.

## 3. Results

### 3.1. Model Fit

**Figure 2:**
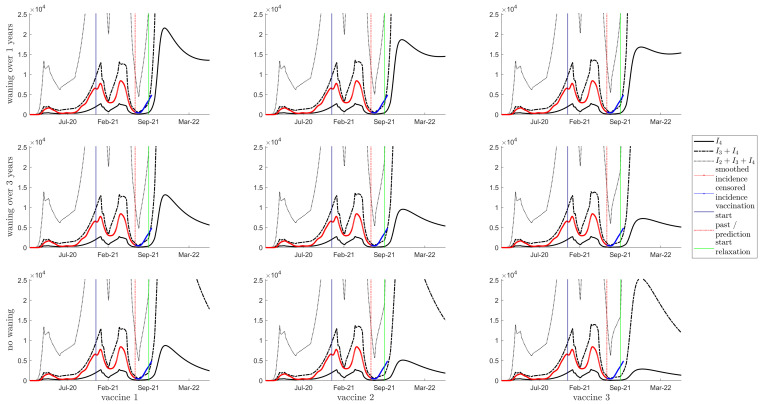
Daily Incidence, with a 20% relaxation of κ starting in September 2021. Showing daily incidence of $I_{4}$ (solid line), $I_{3}+I_{4}$ (dashed line) and $I_{2}+I_{3}+I_{4}$ (dotted line). Smoothed incidence data is shown with a solid red line. The vertical blue, red and green lines indicate the start of vaccination, the beginning of the prediction phase, the start of relaxation, respectively. The top row is waning of immunity by one year between consecutive classes; the middle row is waning of immunity of three years; the bottom row is no waning of immunity. Columns left to right represent vaccines 1 to 3, respectively.

Figure 2 shows the daily incidence of severe, moderate + severe, and mild + moderate + severe infections for the model fitting to 27 June for all nine scenarios. Daily incidence data is shown in red and blue, with the last day of the fitting denoted by the red vertical line. The data indicated by the blue line shows appropriate model trend. We note that the model fit is similar between each of these subplots. We also note that one-year waning seems to match the trend more closely than the other waning rates.

The fitted values of κ are shown in Figure A2 (bottom panel) considering each vaccination and waning scenario. A description of the model parameters and the fitting algorithm is included in the Appendices. In Figure A2 (top panel), we plot the percent reduction in contacts from contact matrix modifications and κ (red +’s) for Vaccine 1 with a waning rate ω=1/year (see Table 1 and dashed blue line in Figure A2 bottom panel).

We note that Figures S1 and S4 are analogous, showing the same fitting results as presented in Figure 2. Large differences between these figures lie only from September 2021, when school opens, and variable increases in κ are implemented. From September 2021 we consider three different scenarios for κ: no predicted change from the last estimated value (Figure S1), a 20% increase in κ (Figure 2), and a 38% increase (Figure S4).

### 3.2. Seroprevalence

**Figure 3:**
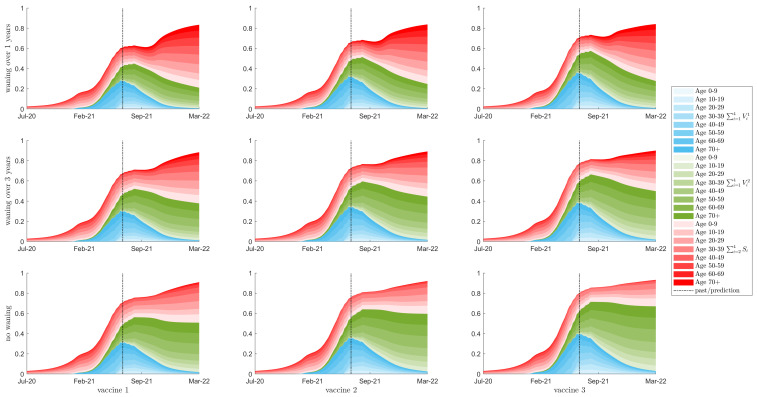
Seroprevalence, with a 20% relaxation of κ starting in September 2021. Showing seroprevalence as a percent of the total population for 10 year age classes, with colour intensity corresponding to age class. The red region is the sum of a susceptible classes that have been exposed to the virus, either from natural infection or through waning from the vaccinated classes. The blue and green regions show the populations of the first and second dose vaccinated classes respectively. The total population with some immunity (the top of the red region) is equal to the vertical sum of the three Blue, Red, and Green regions. The top row is waning of immunity by one year between consecutive classes; the middle row is waning of immunity of three years; the bottom row is no waning of immunity. Columns left to right represent vaccines 1 to 3, respectively. The vertical bar denotes the beginning of the prediction phase.

Given the model fitting above, we can now determine estimates of population seroprevalence. Figure 3, Figures S2 and S5 provide measurements of seroprevalence in the Canadian population given all vaccine types (Table 1) and changes in κ from September 2021 (assuming κ values increase by 20%, no change, and 38%, respectively). These figures show all classes that confer some immunity to SARS-CoV-2, over all age groups. The colours denote the sum of the one-dose vaccinated sub-populations ($\sum_{i}V_{i}^{1}$), the two-dose vaccinated sub-populations ($\sum_{i}V_{i}^{2}$), and the susceptible classes with partial and full immunity ($S_{2}+S_{3}+S_{4}$), over 10-year age groups.The shading is related to the age classes, with lighter to darker coinciding with younger to older ages. In all nine scenarios, 60 to 80% of the Canadian population has some immunity against the pathogen (derived from infection and/or vaccination) by the time schools re-open in September 2021.

While the seroprevalence predicted by our model is higher than that projected by [5], there is little difference in the vaccine derived immunity between our results. The difference thus lies in the estimates of immunity derived from infections. While our model includes age structure, we must note that the system of ordinary differential equations assumes that the population is mixing at a higher level than in reality. It is therefore expected that the model will estimate higher levels of immunity. We note, however, that as vaccination coverage increases and becomes dominant in the population, the difference in seroprevalence estimates from our model and [5] should reduce. We also note that [5] does not include seroreversion (related to waning immunity). Inclusion of seroreversion will boost seroprevalence estimates in their work, so the difference between our results and theirs would reduce further.

**Figure 4:**
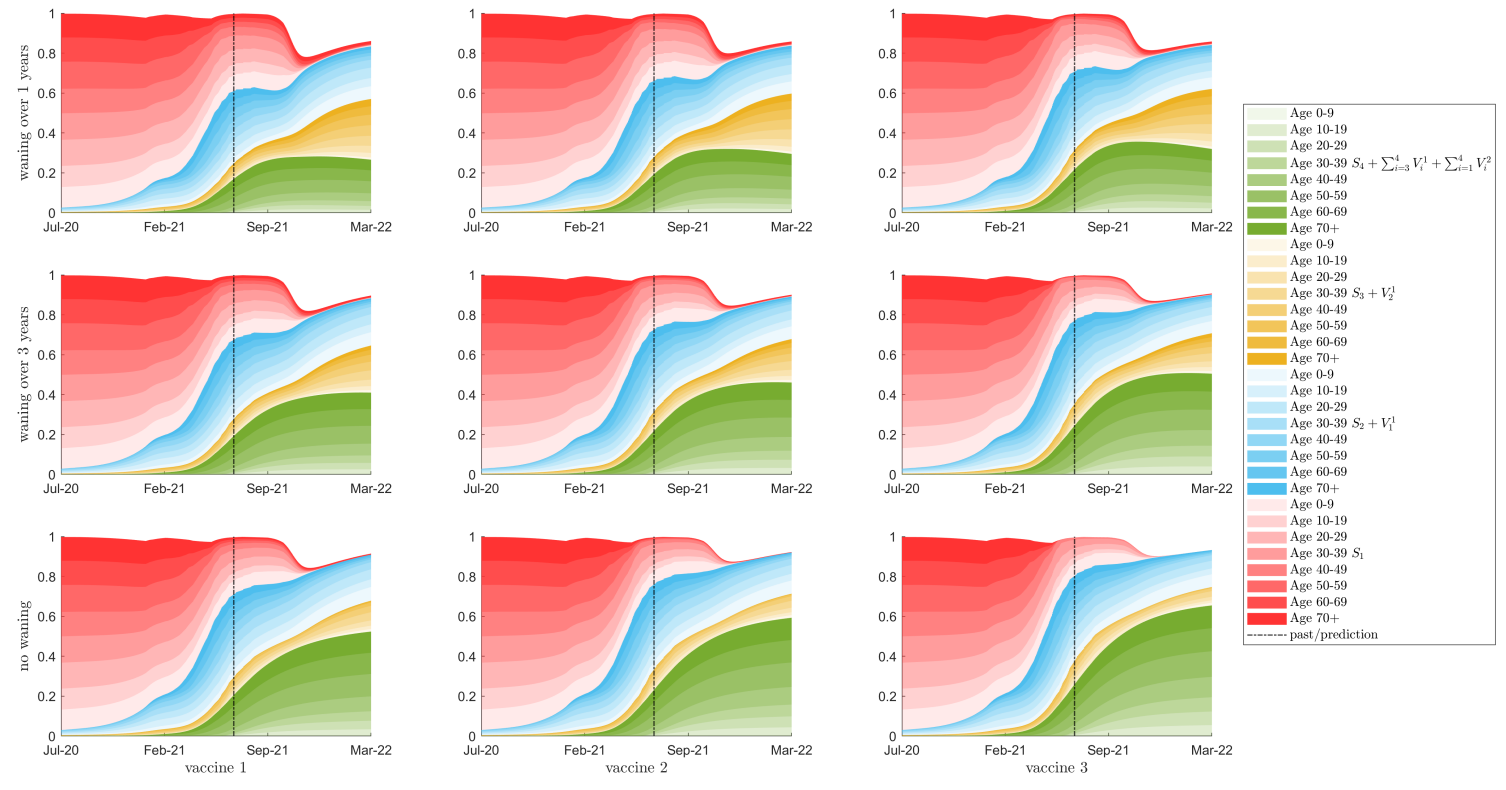
Distribution of Immunity, with a 20% relaxation of κ starting in September 2021. Showing immune status of uninfected individuals as a percent of the total population for 10 year age classes, with colour intensity corresponding to age class. The colours represent the immunity of the population from green, the fully immune population, through yellow and blue to red, the fully susceptible population. The top row is waning of immunity by one year between consecutive classes; the middle row is waning of immunity of three years; the bottom row is no waning of immunity. Columns left to right represent vaccines 1 to 3, respectively. The vertical bar denotes the beginning of the prediction phase.

For interest, in Figure 4, Figures S3 and S6 (assuming increases in κ values of 20%, no change, and 38%, respectively), we plot the corresponding distribution of protection against the virus of the different types of immunity in the population, over 10-year age groups, including no protection ($S_{1}$), some protection ($S_{2}+V_{1}^{1}$), a higher level of protection ($S_{3}+V_{2}^{1}$), and full protection ($S_{4}+\sum_{i=3,4}V_{i}^{1}+\sum_{i=1,\ldots ,3}V_{i}^{2}$). The white area denotes the fraction of the population that resides in the exposed and infected classes $E_{j}^{k}$, $I_{j}$, with *j* (2≤j≤4) and *k* (1≤k≤3).

Figure 3, Figure 4, Figures S2, S3, S5 and S6 show model predictions of the dominant level of immunity for each age class for each of scenario. We note that these outcomes are related to vaccine availability for each age group, model assumptions related to infection induced immunity, and the assumed vaccine acquired immunity characteristics (Table 1 which affect the circulation of the virus in the population. It is obvious in all of these figures that the younger ages, 0–10 year of age, have large levels of susceptibility (light red bars). This is due to the fact that vaccines are not yet available for these ages. Additionally, the mild infections that predominantly occur in these age groups confer low levels of immunity (light blue bars) that can wane quickly back to full susceptibility. The figures also show large levels of susceptibility in age groups 10–29 despite the availability of COVID-19 to 12–29 year olds. Finally, these figures show the effects of waning immunity. Immunity decays from higher immune classes to lower immune classes over time. Given that the early stages of the COVID-19 vaccine rollout in Canada centred on the older age groups, we observe increases in full susceptibility in the older age groups as time since vaccination increases (see dark red bars). Overall, the results in these figures suggest that increased vaccination coverage of age groups 12–29 should be pursued in government vaccination campaigns. Additionally, these results point to the need for a booster dose of vaccine in older age groups.

### 3.3. Herd Immunity

Herd immunity refers to a level of immunity, $p_{c}$, that is needed in a population in order for that population to be resistant to further infection (a small number of infections may occur, but the disease will die out). Briefly, in a population with long-term effective immunity, the herd immunity threshold can be approximated by $p_{c}=1-1/R_{0}$, where $p_{c}$ is the critical fraction of the population that needs to have neutralizing immunity in order for the entire population to be protected, $R_{0}$ is the basic reproduction number of the pathogen in circulation, and homogeneous mixing in the population is assumed. Given a vaccine with vaccine efficacy $0<v_{e}<1$, this approximation can be modified to be $p_{c}=\left(1-1/R_{0}\right)/v_{e}$. We note that with the assumption of homogeneous mixing, this approximation will not provide a best estimate of the herd immunity threshold in age-based mixing models like ours (using contact matrices between age groups, and assuming preferential and proportional mixing), specifically considering different age-specific rates of vaccination [21,22,23,24,25,26,27,28,29]. Additionally, we note that the approximation assumes long-term or life-long immunity to be gained after infection or vaccination. If immunity wanes over time, and if the pathogen evolves, which decays effective immunity against infection over time, this will underestimate the immune population needed to provide herd immunity [29,30]. Nevertheless, we now consider the approximation with homogeneous mixing to estimate a herd immunity threshold for discussion. Currently, with the δ-variant in circulation, $p_{c}=94\%$, given a reproduction number of 6.5 (between 5 and 8 [31]) and a vaccine efficacy of 90% [13,14,15,16,17,18,19]. Our model predicts that 60 to 80% of the Canadian population will have some immunity to SARS-CoV-2 by the end of the vaccination campaign in late Summer 2021. We also observe that approximately 20 to 50% of the population will have neutralizing immunity, depending on the assumed waning rate and the characteristics of the vaccine (see Figure 4, Figures S3 and S6, green-shaded areas). This value of 20 to 50%, from our model (which incorporates age structure, waning immunity from infection and vaccination, and differential immunity gained after infection into our model structure) is far from the estimated herd immunity threshold of 94% (assuming homogeneous mixing and long-term immunity).

We note that benefits of vaccination programs are in their provision of protection from infection, but also in their protection from severe disease. Given 20–50% neutralizing immunity is not sufficient to protect the population from further infection, we now focus our attention to identifying populations that are at risk of severe infections in COVID-19 resurgence, given waning immunity from vaccination and infection, and protective capacities of immunity against infection and severe disease.

### 3.4. Resurgence

Figure 2, Figures S1 and S4 show no significant difference between model fits, and through the summer trend, but we do observe significant divergence past September, coinciding with the reopening of schools (Figure S1), and increases in κ by 20% and 38% (Figure 2 and Figure S4, respectively). When seroprevalence is high, corresponding to lower rates of waning immunity, we see that resurgence in infections, denoted by the white space, occurs in a reduced fashion. However, over all scenarios but one (when immunity does not wane, and vaccine protection is high, bottom right subplot), the model projects that the Fall 2021 resurgence will reach levels far greater than any wave of COVID-19 infection previously experienced.

We note that the resurgence in Fall 2021 is mainly driven by the virus circulation in Summer 2021. Figure 2 shows that our model captures the general trend in increased COVID-19 transmission in Summer 2021, and therefore, the Fall resurgence levels projected here are reasonable projections. Model results do however also show that if relaxation in July 2021 had not occurred, limiting the transmission of the delta VOC, resurgence would be greatly delayed and would have much lower infection levels in Summer and Fall 2021 (results not shown).

**Figure 5:**
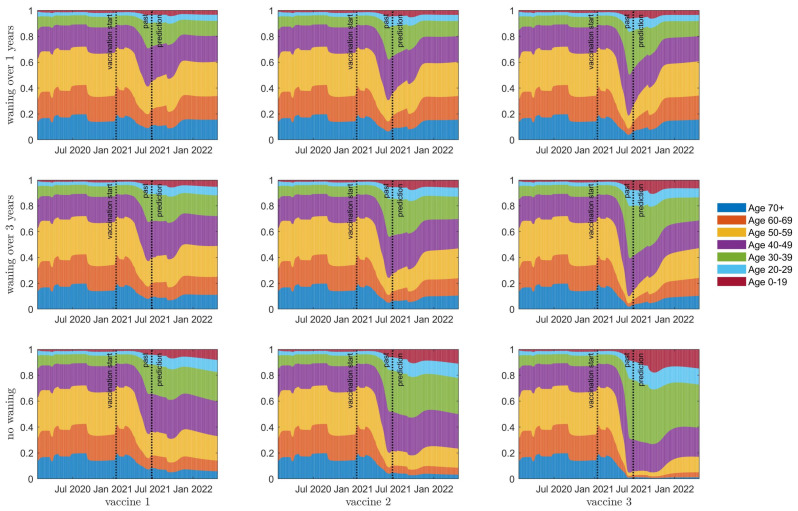
Age structure of daily incidence of severe disease, $I_{4}$, over 7 different age groups (see legend and corresponding colours). The top row is waning of immunity by one year between consecutive classes; the middle row is waning of immunity of three years; the bottom row is no waning of immunity. Columns left to right represent vaccines 1 to 3, respectively.

Individuals who received the vaccine early in the vaccination program will likely have experienced some effects of waning immunity by September 2021 (see Figure 3, Figure 4, Figures S2, S3, S5 and S6). Resurgence in infections will thus be stronger in these age groups. Figure 5 plots the proportion of daily $I_{4}$ infection incidence by age for each vaccine scenario, when school is reopened in September and κ is increased by 20%. Here, we see that resurgent cases are predominantly observed in age groups 50+ or 40+ when immunity wanes over 1 year or 3 years between consecutive immune classes, respectively (top and middle rows, respectively). Considering that COVID-19 infection fatality rates increase by age [5], we suggest that a vaccine booster campaign be considered in older age groups.

**Figure A1:**
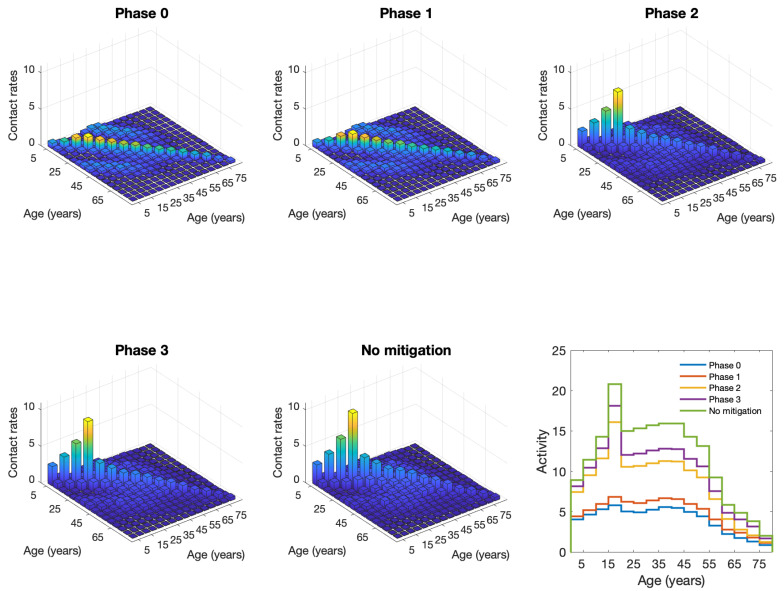
Contact rates and activity under different phases of mitigation. Bar plots show contact rates between age groups under different mitigation strategies, with compliance (*k*) assumed to be 100%. Taller bars, by subplot, have lighter colors. All bar plots have identical y-axis scale for comparison (but are not identical in colour scale). The total activity is shown in the line plot (bottom right).

**Figure A2:**
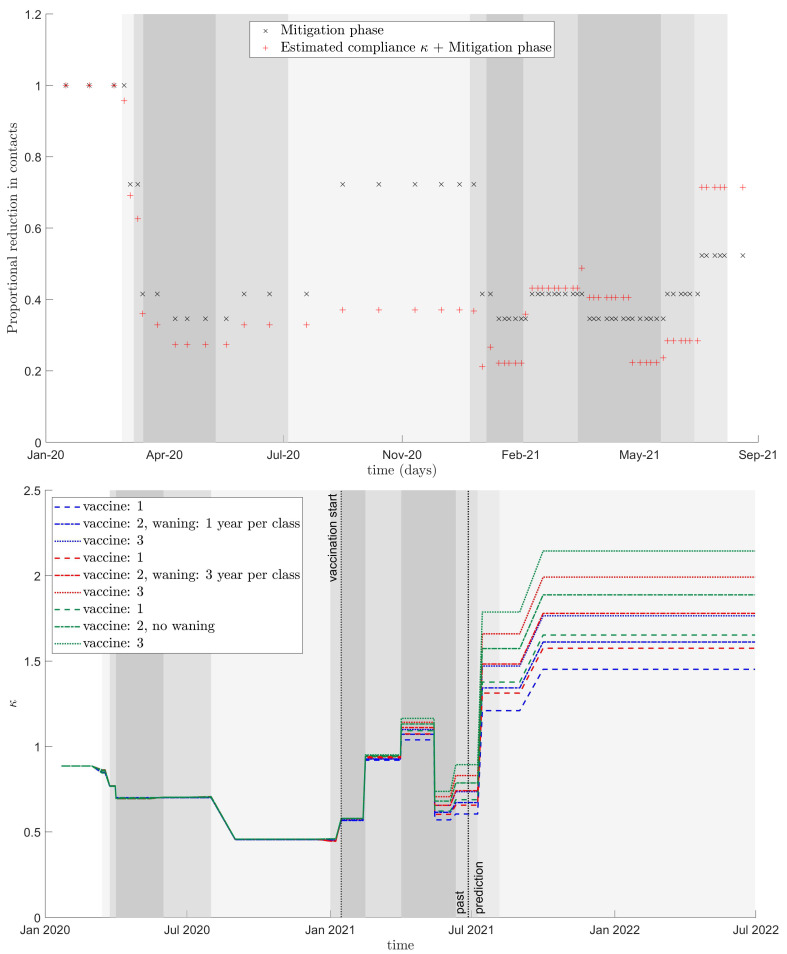
Changes in contact levels over time. (top panel) Average overall contacts compared to no mitigation from modified contact matrices (x’s) plus parameter κ (red +’s). (bottom panel) Value of parameter κ from the model fit (before black vertical line), and into the future (after black vertical line). A 20% increase in κ in September 2021 is shown here. In both panels, mitigation phases are shown with background shading where darker shading represents more restrictive mitigation, see Table A3 and Table A4 for details.

The degree of resurgence is affected by increases in κ and school re-opening. Given the higher levels of average daily contacts in younger age groups (see Figure A1, bottom right panel), and given that there is no vaccine yet approved for these ages, we recommend continued use of protective measures in schools, including mask wearing and social distancing. We also recommend that vaccination begin for these ages immediately after a vaccine is approved.

## 4. Discussion

Our model predicts that 60 to 80% of the Canadian population will have some immunity to SARS-CoV-2 by the end of the vaccination campaign in late Summer 2021. The population is vulnerable to virus resurgence, however, given the relaxation of non-pharmaceutical measures over Summer 2021 which allowed spread of the delta VOC. Model results pinpoint the need for increased vaccine coverage in ages 12–29, and booster doses in ages 50+.

The mathematical modelling study presented here is the first to address quantification of COVID-19 seroprevelance and distributions of immunity in a population. The model includes immunity gains from infection and vaccination, and also includes immunity decline due to waning. It is structured by 5-year age groups. As immunity is lost, model results can be used to identify age groups requiring vaccine booster campaigns, or increased vaccination coverage. It can also be used to determine the age groups most affected by COVID-19 resurgence.

The timing and severity of any resurgence is sensitive to the distribution of immunity. It is also sensitive to the introduction of VOCs, the cessation of personal protective measures and public health mitigation. This sensitivity is highlighted through the comparison of Figure 2 and Figure S4. We note that the possible effects of imported cases are not included in our model, and therefore cannot be gauged as to their effect on COVID-19 resurgence. This will require further evaluation, however, if the virus remains endemic in the population, importation is unlikely to substantially alter the model outputs according to recent modelling by members of our group [32].

## Figures and Tables

**Table 1:** Vaccine efficacy. Three considered vaccine’s first and two-dose efficacy against infection and against severe disease.

	Against Infection		Against Severe Disease
	Two Doses	First Dose	
Vaccine 1	70%	50%	75%
Vaccine 2	80%	70%	80%
Vaccine 3	90%	70%	92%

## Data Availability

Canadian incidence data is taken from an online data portal. Please see [7] for details.

## Supplementary Materials

The following are available online at https://www.mdpi.com/article/10.3390/vaccines10010017/s1, Figure S1: Daily Incidence, with a no relaxation of κ. Showing daily incidence of $I_{4}$ (solid line), $I_{3}+I_{4}$ (dashed line) and $I_{2}+I_{3}+I_{4}$ (dotted line). Smoothed incidence data is shown with a solid red line. The vertical blue, red and green lines indicate the start of vaccination, the beginning of the prediction phase, the start of relaxation, respectively. The top row is waning of immunity by one year between consecutive classes; the middle row is waning of immunity of three years; the bottom row is no waning of immunity. Columns left to right represent vaccines 1 to 3, respectively. Figure S2: Seroprevalence, with no relaxation of κ. Showing seroprevalence as a percent of the total population for 10 year age classes, with colour intensity corresponding to age class. The red region is the sum of a susceptible classes that have been exposed to the virus, either from natural infection or through waning from the vaccinated classes. The blue and green regions show the populations of the first and second dose vaccinated classes respectively. The total population with some immunity (the top of the red region) is equal to the vertical sum of the three Blue, Red, and Green regions. The top row is waning of immunity by one year between consecutive classes; the middle row is waning of immunity of three years; the bottom row is no waning of immunity. Columns left to right represent vaccines 1 to 3, respectively. The vertical bar denotes the beginning of the prediction phase. Figure S3: Distribution of Immunity, with no relaxation of κ. Showing immune status of uninfected individuals as a percent of the total population for 10 year age classes, with colour intensity corresponding to age class. The colours represent the immunity of the population from green, the fully immune population, through yellow and blue to red, the fully susceptible population. The top row is waning of immunity by one year between consecutive classes; the middle row is waning of immunity of three years; the bottom row is no waning of immunity. Columns left to right represent vaccines 1 to 3, respectively. The vertical bar denotes the beginning of the prediction phase. Figure S4: Daily Incidence, with a 38% relaxation of κ starting in September 2021. Showing daily incidence of $I_{4}$ (solid line), $I_{3}+I_{4}$ (dashed line) and $I_{2}+I_{3}+I_{4}$ (dotted line). Smoothed incidence data is shown with a solid red line. The vertical blue, red and green lines indicate the start of vaccination, the beginning of the prediction phase, the start of relaxation, respectively. The top row is waning of immunity by one year between consecutive classes; the middle row is waning of immunity of three years; the bottom row is no waning of immunity. Columns left to right represent vaccines 1 to 3, respectively. Figure S5: Seroprevalence, with a 38% relaxation of κ starting in September 2021. Showing seroprevalence as a percent of the total population for 10 year age classes, with colour intensity corresponding to age class. The red region is the sum of a susceptible classes that have been exposed to the virus, either from natural infection or through waning from the vaccinated classes. The blue and green regions show the populations of the first and second dose vaccinated classes respectively. The total population with some immunity (the top of the red region) is equal to the vertical sum of the three Blue, Red, and Green regions. The top row is waning of immunity by one year between consecutive classes; the middle row is waning of immunity of three years; the bottom row is no waning of immunity. Columns left to right represent vaccines 1 to 3, respectively. The vertical bar denotes the beginning of the prediction phase. Figure S6: Distribution of Immunity, with a 38% relaxation of κ starting in September 2021. Showing immune status of uninfected individuals as a percent of the total population for 10 year age classes, with colour intensity corresponding to age class. The colours represent the immunity of the population from green, the fully immune population, through yellow and blue to red, the fully susceptible population. The top row is waning of immunity by one year between consecutive classes; the middle row is waning of immunity of three years; the bottom row is no waning of immunity. Columns left to right represent vaccines 1 to 3, respectively. The vertical bar denotes the beginning of the prediction phase.

## Appendix A

### Appendix A.1. Model Equations

Our model tracks age, infection and immune status. Susceptible individuals of status *i* and age *k* are denoted by $S_{ik}$; similarly, infectious individuals by $I_{ik}$. Infected but not-yet-infectious individuals of immune status *i*, age *k* and stage *j* are denoted by $E_{ik}^{j}$. Vaccinated individuals of initial immune status *i*, age *k* and dose *k* are denoted by $V_{ik}^{j}$. Parameter descriptions are found in Table A1. The system of ODEs for age group *k* is given by the following set of equations:

Susceptible compartments:

$$\begin{array}{cc} \frac{d}{dt}S_{1k} & =-\sum_{j=2}^{4}p_{1k}^{j}\Lambda_{1k}S_{1k}+\omega_{2k}S_{2k}-\sigma_{1k}^{1}\left(t\right)\rho S_{1k}+\omega_{2k}V_{1k}^{1},\\ \frac{d}{dt}S_{2k} & =-\sum_{j=2}^{4}p_{2k}^{j}\Lambda_{2k}S_{2k}+\omega_{3k}S_{3k}-\omega_{2k}S_{2k}-\sigma_{2k}^{1}\left(t\right)\rho S_{2k}+\gamma_{2k}I_{2k}+\omega_{3k}V_{2k}^{1},\\ \frac{d}{dt}S_{3k} & =-\sum_{j=3}^{4}p_{3k}^{j}\Lambda_{3k}S_{3k}+\omega_{4k}S_{4k}-\omega_{3k}S_{3k}-\sigma_{3k}^{1}\left(t\right)\rho S_{3k}+\gamma_{3k}I_{3k}+\omega_{4k}\left(\sum_{j=3}^{4}V_{jk}^{1}+\sum_{j=1}^{4}V_{jk}^{2}\right),\\ \frac{d}{dt}S_{4k} & =-\omega_{4k}S_{4k}-\sigma_{4k}^{1}\left(t\right)\rho S_{4k}+\gamma_{4k}I_{4k}, \end{array}$$

Vaccinated compartments:

$$\begin{array}{cc} \frac{d}{dt}V_{1k}^{1} & =\sigma_{1k}^{1}\left(t\right)\rho S_{1k}-\sigma_{1k}^{2}\left(t\right)\rho V_{1k}^{1}-\sum_{j=2}^{4}p_{2k}^{j}\Lambda_{2k}V_{1k}^{1}-\omega_{2k}V_{1k}^{1},\\ \frac{d}{dt}V_{2k}^{1} & =\sigma_{2k}^{1}\left(t\right)\rho S_{2k}-\sigma_{2k}^{2}\left(t\right)\rho V_{2k}^{1}-\sum_{j=3}^{4}p_{3k}^{j}\Lambda_{3k}V_{2k}^{1}-\omega_{3k}V_{2k}^{1},\\ \frac{d}{dt}V_{3k}^{1} & =\sigma_{3k}^{1}\left(t\right)\rho S_{3k}-\sigma_{3k}^{2}\left(t\right)\rho V_{3k}^{1}-\omega_{4k}V_{3k}^{1},\\ \frac{d}{dt}V_{4k}^{1} & =\sigma_{4k}^{1}\left(t\right)\rho S_{4k}-\sigma_{4k}^{2}\left(t\right)\rho V_{4k}^{1}-\omega_{4k}V_{4k}^{1},\\ \frac{d}{dt}V_{1k}^{2} & =\sigma_{1k}^{2}\left(t\right)\rho V_{1k}^{1}-\omega_{4k}V_{1k}^{2},\\ \frac{d}{dt}V_{2k}^{2} & =\sigma_{2k}^{2}\left(t\right)\rho V_{2k}^{1}-\omega_{4k}V_{2k}^{2},\\ \frac{d}{dt}V_{3k}^{2} & =\sigma_{3k}^{2}\left(t\right)\rho V_{3k}^{1}-\omega_{4k}V_{3k}^{2},\\ \frac{d}{dt}V_{4k}^{2} & =\sigma_{4k}^{2}\left(t\right)\rho V_{4k}^{1}-\omega_{4k}V_{4k}^{2}, \end{array}$$

Infected compartments:

$$\begin{array}{cc} \frac{d}{dt}E_{2k}^{1} & =p_{1k}^{2}\Lambda_{1k}S_{1k}+p_{2k}^{2}\Lambda_{2k}S_{2k}+p_{2k}^{2}\Lambda_{2k}V_{1k}^{1}-\zeta_{2k}^{1}E_{2k}^{1},\\ \frac{d}{dt}E_{3k}^{1} & =p_{1k}^{3}\Lambda_{1k}S_{1k}+p_{2k}^{3}\Lambda_{2k}S_{2k}+p_{2k}^{3}\Lambda_{2k}V_{1k}^{1}+p_{3k}^{3}\Lambda_{3k}S_{3k}+p_{3k}^{3}\Lambda_{3k}V_{2k}^{1}-\zeta_{3k}^{1}E_{3k}^{1},\\ \frac{d}{dt}E_{4k}^{1} & =p_{1k}^{4}\Lambda_{1k}S_{1k}+p_{2k}^{4}\Lambda_{2k}S_{2k}+p_{2k}^{4}\Lambda_{2k}V_{1k}^{1}+p_{3k}^{4}\Lambda_{3k}S_{3k}+p_{3k}^{4}\Lambda_{3k}V_{2k}^{1}-\zeta_{4k}^{1}E_{4k}^{1},\\ \frac{d}{dt}E_{2k}^{2} & =\zeta_{2k}^{1}E_{2k}^{1}-\zeta_{2k}^{2}E_{2k}^{2},\\ \frac{d}{dt}E_{3k}^{2} & =\zeta_{3k}^{1}E_{3k}^{1}-\zeta_{3k}^{2}E_{3k}^{2},\\ \frac{d}{dt}E_{4k}^{2} & =\zeta_{4k}^{1}E_{4k}^{1}-\zeta_{4k}^{2}E_{4k}^{2},\\ \frac{d}{dt}E_{2k}^{3} & =\zeta_{2k}^{2}E_{2k}^{2}-\zeta_{2k}^{3}E_{2k}^{3},\\ \frac{d}{dt}E_{3k}^{3} & =\zeta_{3k}^{2}E_{3k}^{2}-\zeta_{3k}^{3}E_{3k}^{3},\\ \frac{d}{dt}E_{4k}^{3} & =\zeta_{4k}^{2}E_{4k}^{2}-\zeta_{4k}^{3}E_{4k}^{3},\\ \frac{d}{dt}I_{2k} & =\zeta_{2k}^{3}E_{2k}^{3}-\gamma_{2k}I_{2k}-\delta_{2k}I_{2k},\\ \frac{d}{dt}I_{3k} & =\zeta_{3k}^{3}E_{3k}^{3}-\gamma_{3k}I_{3k}-\delta_{3k}I_{3k},\\ \frac{d}{dt}I_{4k} & =\zeta_{4k}^{3}E_{4k}^{3}-\gamma_{4k}I_{4k}-\delta_{4k}I_{41}, \end{array}$$

where for 1≤k≤N,

$$\begin{array}{cc} \Lambda_{ik}\left(t\right) & =\alpha_{ik}A_{k}\lambda_{ik}\left(t\right),\\ \lambda_{ik}\left(t\right) & =\sum_{m=1}^{N}c_{km}\frac{\sum_{j=2}^{4}\beta_{jm}I_{jm}\left(t\right)}{\sum_{j=1}^{4}T_{jm}\left(t\right)},\\ T_{jm}\left(t\right) & =S_{jm}\left(t\right)+V_{jm}^{1}\left(t\right)+V_{jm}^{2}\left(t\right)+E_{jm}^{1}\left(t\right)+E_{jm}^{2}\left(t\right)+E_{jm}^{3}\left(t\right)+I_{jm}\left(t\right). \end{array}$$

### Appendix A.2. Reproduction Number

The basic reproduction number is calculated using the Next Generation Method. It is calculated numerically, due to the dimensionality of the model system. Briefly, we determine the spectral radius of the matrix $G=FV^{-1}$, where matrix *F* is related to·new infections, and matrix *V* is related to transfers between classes infected classes. We refer the reader to [24,33,34,35] for important references on this method.

We assume a basic reproduction number of $R_{0}=2.6$ [36]. This is used to determine κ in the model fitting routine.

## Appendix B. Parameters

**Table A1:** Parameter definitions for the ODE model.

Parameter	Definition
$\alpha_{in}$	susceptibility of individuals from $S_{in}$ (*i* immunity status, *n* age group)
$\beta_{jm}$	infectivity of infected individuals from $I_{jm}$ (*j* immunity status, *m* age group)
$\gamma_{jm}$	recovery rate of infected individuals from $I_{jm}$ (*j* immunity status, *m* age group)
$\zeta_{jm}^{k}$	rates of progress through the pre-infectious period of infeciton (*i* immunity status, *n* age group, *k* stage)
$\delta_{jm}$	disease-induced mortality rate of infected individuals from $I_{jm}$ (*j* immunity status, *m* age group)
$\omega_{in}$	waning rate of immunity of individuals from $S_{in}$ (*i* immunity status, *n* age group)
$p_{in}^{j}$	proportion of $S_{i}$ going to $I_{j}$ upon infection with i=1,2,3 and j=2,3,4 (i,j immunity status, *n* age group)
$\rho_{in}$	vaccine efficacy (*i* immunity status, *n* age group)
$\sigma_{k}$	vaccination rate for first k=1 and second k=2 dose
$A_{n}$	per capita activity counts of individuals in age group *n*
$c_{an}$	mixing matrix between individuals in age group *a* and age group *n*

Susceptibility: assume $\alpha_{1n}=1$, $\alpha_{2n}=\frac{2}{3}$, and $\alpha_{3n}=\frac{1}{3}$ for any age group *n*; We assume that susceptibility decreases with increasing immunity status, but does not depend on age.

Infectivity: $\beta_{3n}=\beta =0.08$, $\beta_{2n}=0.5\beta$, $\beta_{4n}=0.1\beta$ for any age group *n*; Values are determined by fitting the model $R_{0}$ value to 2.6 [36]; We assume that infectivity varies by severity of infection and severity of disease—milder infections, with lower viral load, have lower infectivity, and more severe infections will have lower infectivity as such individuals will stay home, or will go to hospital.

Recovery: $\gamma_{1n}<\gamma_{2n}<\gamma_{3n}$, with $\gamma_{1n}=\frac{1}{5}$, $\gamma_{2n}=\frac{1}{10}$, $\gamma_{3n}=\frac{1}{15}$ for any age group *n*; Recovery is assumed to depend on on disease severity, with milder disease associated with shorter infectious periods.

Exposed period: We assume an erlang distribution, erlang (3,2/3), with mean of 3 days.

Waning Immunity: $\omega_{in}=\omega =\frac{1}{yr*365}$ for i=1,2,3 and any age group *n*; We assume immunity gained from infection or vaccination lasts on average 1 to 3 years between successive immunity stages and is independent of age and immunity status. This assumption is varied in the current manuscript, and we also include model outcomes when it is assumed that immunity does not wane.

Probability of severe infection: see Table A2; We assume that severity of infection is associated with the prevalence of comorbidities in each age group. Precise values are determined from [9].

**Table A2:** Proportion of susceptible individuals from $S_{in}$ going to $I_{j}$ infectious class.

Age Group	$p_{in}^{\left(2\right)}$	$p_{in}^{\left(3\right)}$	$p_{in}^{\left(4\right)}$
0–4	0.979187625	0.019532353	0.001280022
5–9	0.970340674	0.02795418	0.001705146
10–14	0.971354725	0.026962603	0.001682673
15–19	0.963790465	0.033836424	0.002373111
20–24	0.94736408	0.048618385	0.004017534
25–29	0.923743993	0.069289774	0.006966234
30–34	0.897203802	0.09151371	0.011282488
35–39	0.865605311	0.116760519	0.01763417
40–44	0.806984827	0.162542801	0.030472373
45–49	0.756587998	0.197861718	0.045550284
50–54	0.690245134	0.241501371	0.068253495
55–59	0.600190415	0.296345592	0.103463993
60–64	0.503245046	0.346804634	0.14995032
65–69	0.409505408	0.383960071	0.206534521
70–74	0.324664092	0.404030163	0.271305745
75+	0.215150255	0.373779207	0.411070537

Vaccination: the vaccination rate of the first dose is determined by the coverage detailed in the NACI document. The rate is determined each week, for particular age groups, following the NACI schedule. Vaccine efficacy for each dose is assumed to be 0.9. Individuals receive a second dose $N_{d}$ days following the first dose and acquire immunity after an additional 14 days. This is determined by vaccination guidelines in Canada [8].

Demographics: Given the short period of examination, we assume the absence of birth, natural mortality and aging. In the current study, we ignore disease induced death.

Contacts between age groups: Perturbations of the contact matrices are implemented to reflect public health mitigation strategies over time. A summary of the perturbations is given in Table A3 and Table A4. A graphical depiction of each public health mitigation phase listed in Table A3 and Table A4 is shown in Figure A1.

**Table A3:** Contact mitigation phase description (% reduction in contacts).

Phase	School Contacts	Other Contacts	Work Contacts
**0**	95%	90% under 65, 95% over 65	75% under 65, 95% over
**1**	95%	75%	70% under 65, 95% over
**2**	95%	40% under 65, 65% over	70% under 65, 95% over
**3**	15% under 20, 25% over	40% under 65, 65% over	35% under 65, 95% over
**no mitigation**	0%	0%	0%

**Table A4:** Contact mitigation phase.

Start	End	Phase
2020-02-05	2020-03-18	no mitigation
2020-03-18	2020-03-31	3
2020-03-31	2020-04-25	1
2020-04-25	2020-06-19	0
2020-06-19	2020-09-01	1
2020-09-01	2021-01-08	3
2021-01-08	2021-01-22	1
2021-01-22	2021-02-19	0
2021-02-19	2021-04-09	1
2021-04-09	2021-05-14	0
2021-05-14	2021-07-15	1
2021-07-15	2021-09-01	2
2021-09-01	2021-12-31	3
2021-09-01	2021-12-31	3

## Appendix C. Model Fitting

The model parameter κ was fit to daily incidence data over monthly windows using a least squares objective function. The objective function included a regularization hyper-parameter which penalized variation in κ to control over fitting. The objective function was minimized by comparison against the model solution for $\frac{3}{5}I_{3}+I_{4}$ using the genetic algorithm as implemented in MATLAB. Convergence of the solution was confirmed by analyzing the the variance of the final population. The final fit for each scenario was fit for all time windows and independently of other scenarios. All additional parameters where informed by literature and chosen such that the basic reproduction number was 2.9 and are detailed above in Appendix B.
