# Supplementary material for: COVID-19 Seroprevalence in Canada Modelling Waning and Boosting COVID-19 Immunity in Canada a Canadian Immunization Research Network Study
Source: Vaccines (Basel). 2021 Dec 23;10(1):17. doi: 10.3390/vaccines10010017 (PMC8779812; doi:10.3390/vaccines10010017)
Supplement: Supplementary file 1 [file vaccines-10-00017-s001.zip › vaccines-1415074-supplementary.pdf]

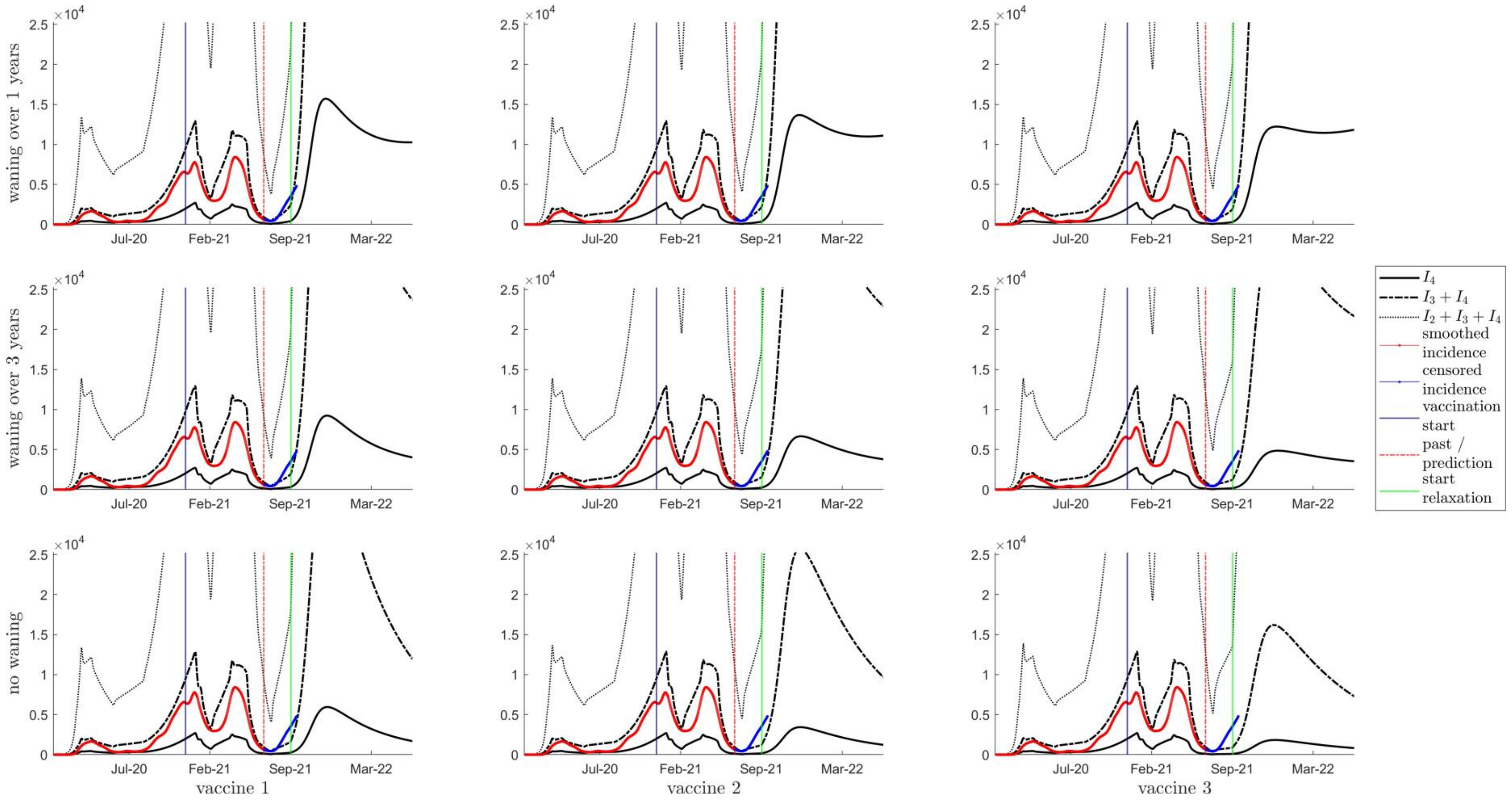

Figure S1: Figure S1: Daily Incidence, with a no relaxation of k. Showing daily incidence of I4 (solid line), I3 + I4 (dashed line) and I2 + I3 + I4 (dotted line). Smoothed incidence data is shown with a solid red line. The vertical blue, red and green lines indicate the start of vaccination, the beginning of the prediction phase, the start of relaxation, respectively. The top row is waning of immunity by one year between consecutive classes; the middle row is waning of immunity of three years; the bottom row is no waning of immunity. Columns left to right represent vaccines 1 to 3, respectively.

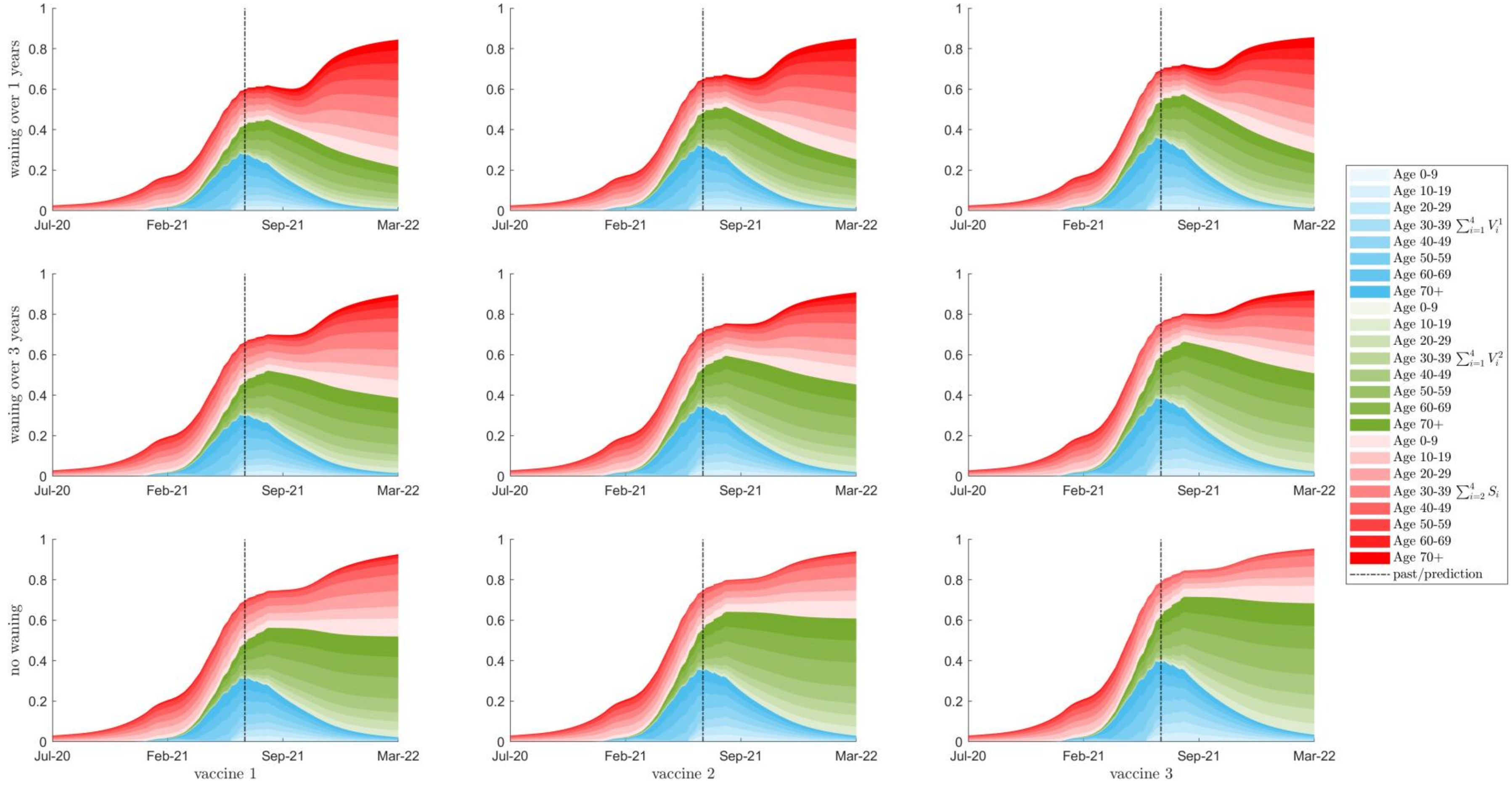

Figure S2: Seroprevalence, with no relaxation of k. Showing seroprevalence as a percent of the total population for 10 year age classes, with colour intensity corresponding to age class. The red region is the sum of a susceptible classes that have been exposed to the virus, either from natural infection or through waning from the vaccinated classes. The blue and green regions show the populations of the first and second dose vaccinated classes respectively. The total population with some immunity (the top of the red region) is equal to the vertical sum of the three Blue, Red, and Green regions. The top row is waning of immunity by one year between consecutive classes; the middle row is waning of immunity of three years; the bottom row is no waning of immunity. Columns left to right represent vaccines 1 to 3, respectively. The vertical bar denotes the beginning of the prediction phase.

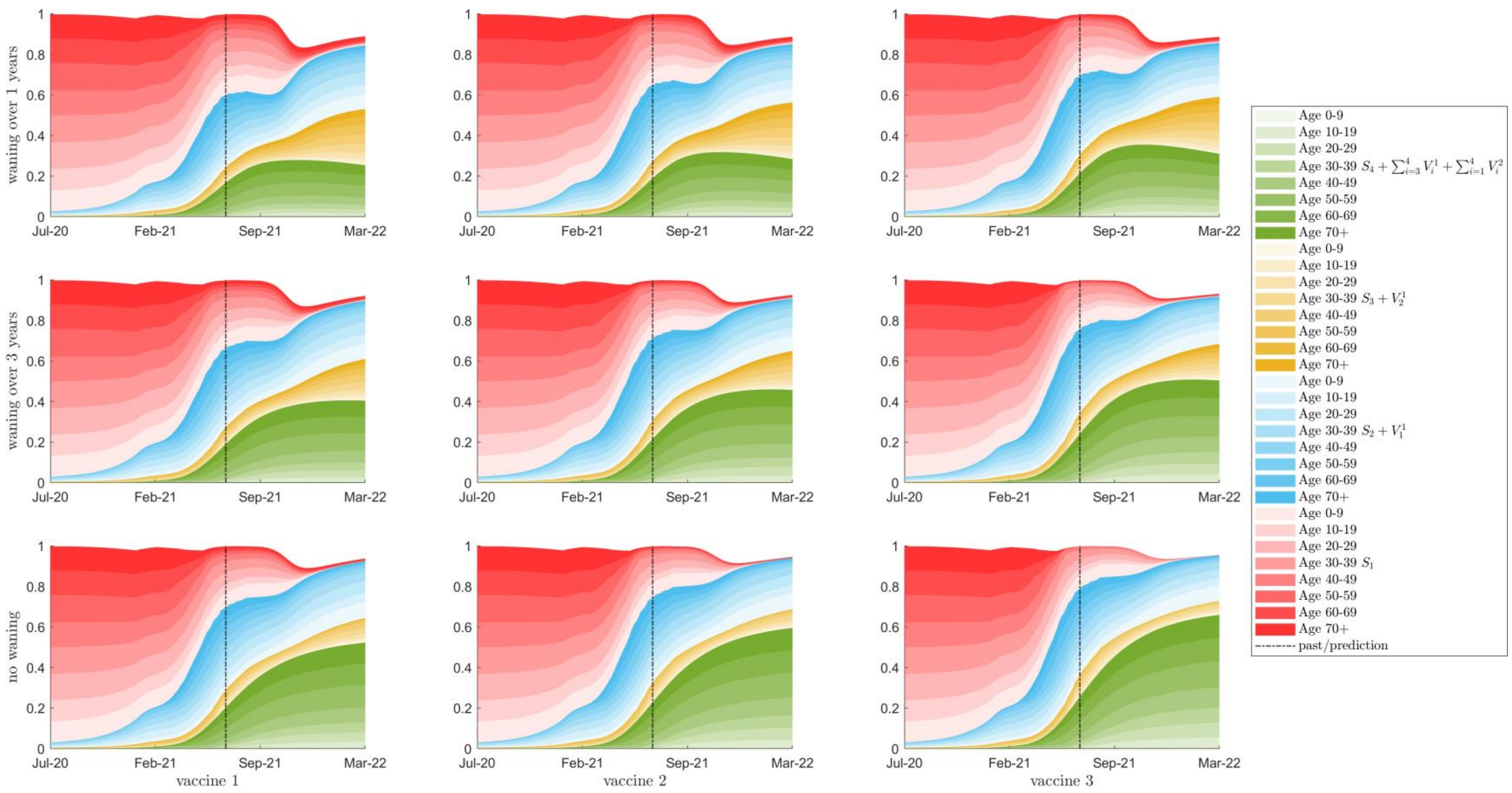

Figure S3: Distribution of Immunity, with no relaxation of k. Showing immune status of uninfected individuals as a percent of the total population for 10 year age classes, with colour intensity corresponding to age class. The colours represent the immunity of the population from green, the fully immune population, through yellow and blue to red, the fully susceptible population. The top row is waning of immunity by one year between consecutive classes; the middle row is waning of immunity of three years; the bottom row is no waning of immunity. Columns left to right represent vaccines 1 to 3, respectively. The vertical bar denotes the beginning of the prediction phase.

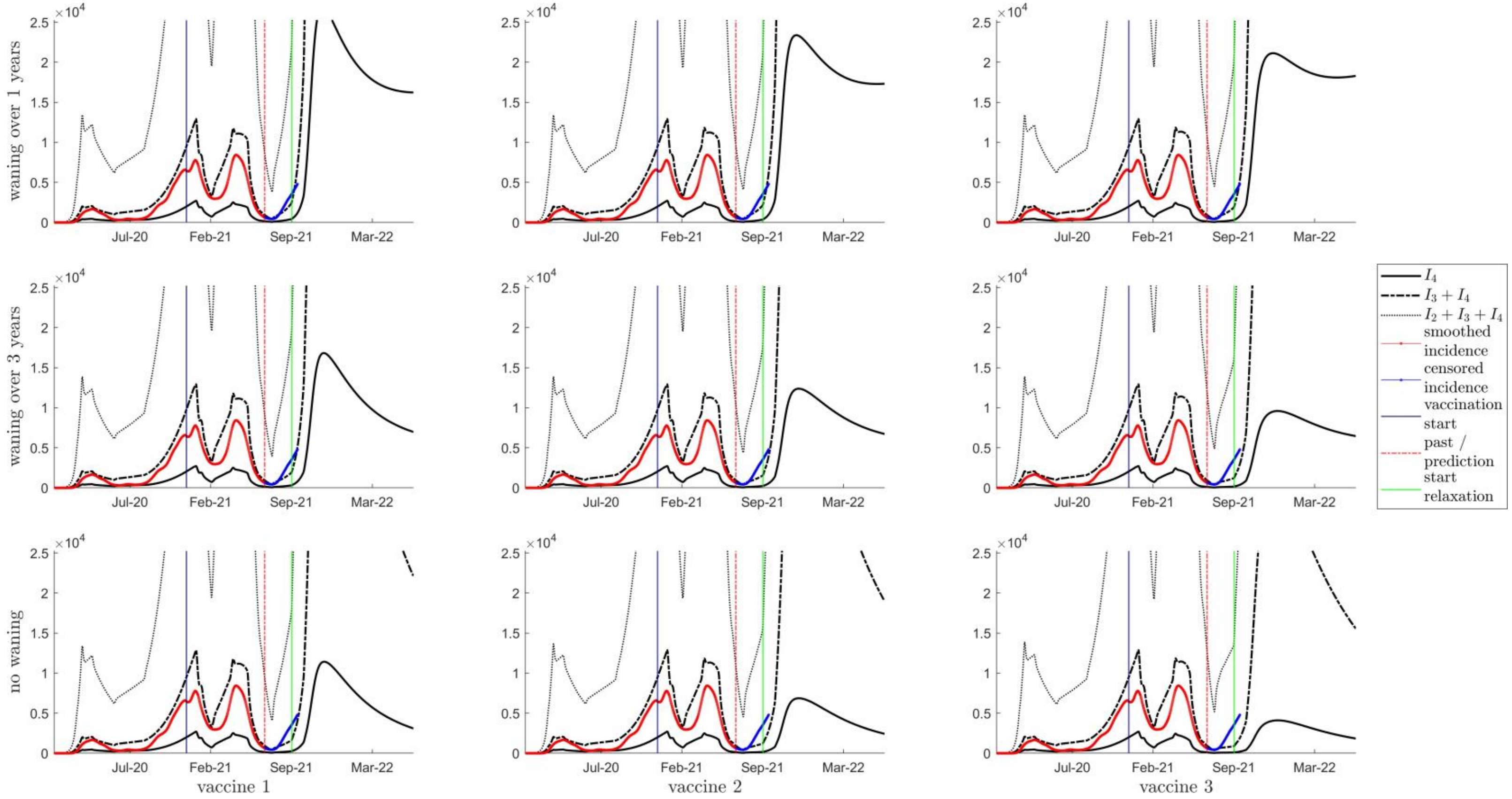

Figure S4 Daily Incidence, with a 38% relaxation of k starting in September 2021. Showing daily incidence of I4 (solid line), I3 + I4 (dashed line) and I2 + I3 + I4 (dotted line). Smoothed incidence data is shown with a solid red line. The vertical blue, red and green lines indicate the start of vaccination, the beginning of the prediction phase, the start of relaxation, respectively. The top row is waning of immunity by one year between consecutive classes; the middle row is waning of immunity of three years; the bottom row is no waning of immunity. Columns left to right represent vaccines 1 to 3, respectively.

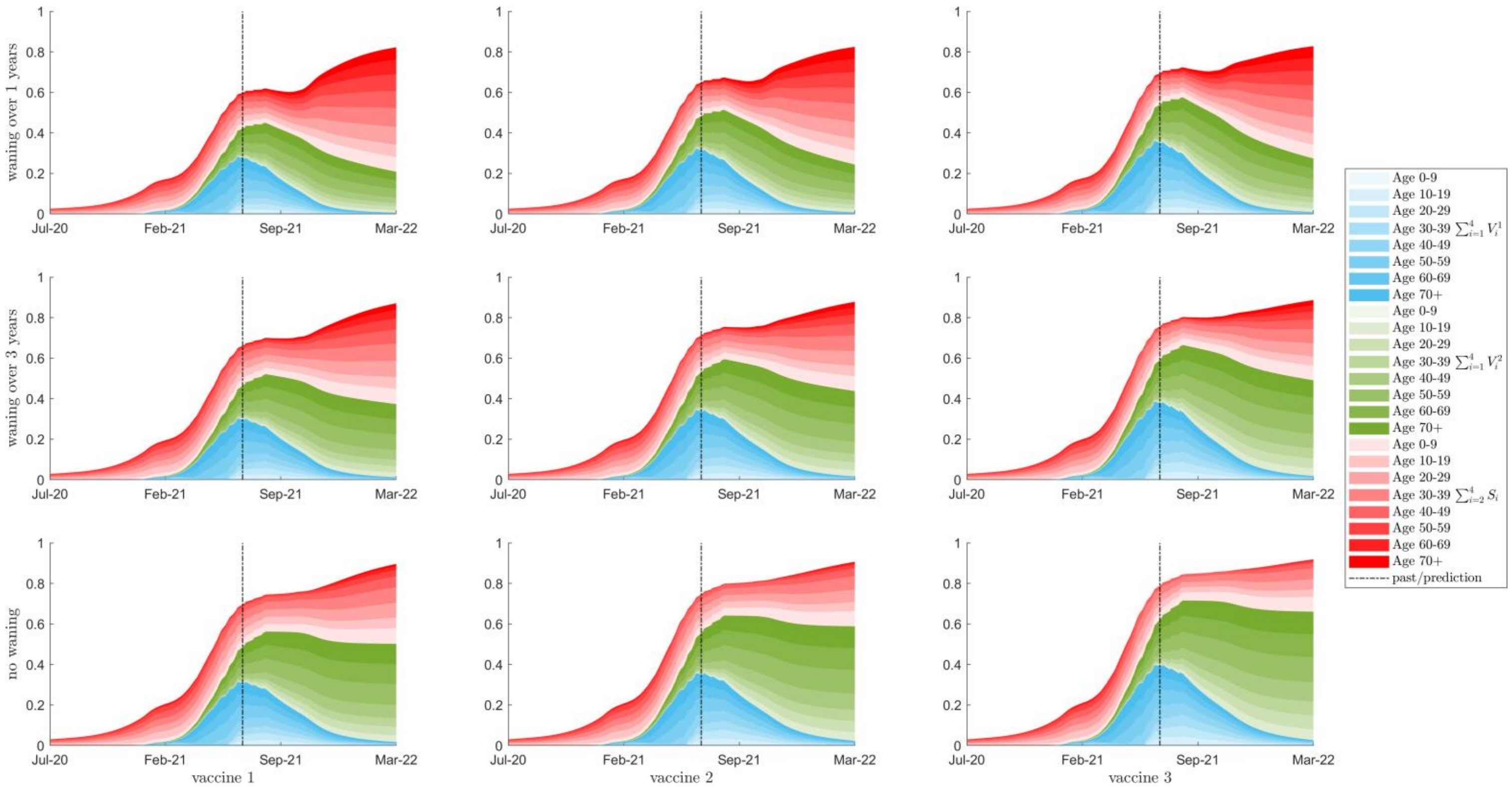

Figure S5: Seroprevalence, with a 38% relaxation of k starting in September 2021. Showing seroprevalence as a percent of the total population for 10 year age classes, with colour intensity corresponding to age class. The red region is the sum of a susceptible classes that have been exposed to the virus, either from natural infection or through waning from the vaccinated classes. The blue and green regions show the populations of the first and second dose vaccinated classes respectively. The total population with some immunity (the top of the red region) is equal to the vertical sum of the three Blue, Red, and Green regions. The top row is waning of immunity by one year between consecutive classes; the middle row is waning of immunity of three years; the bottom row is no Vaccines 2021, 1, 0 12 of 19 waning of immunity. Columns left to right represent vaccines 1 to 3, respectively. The vertical bar denotes the beginning of the prediction phase.

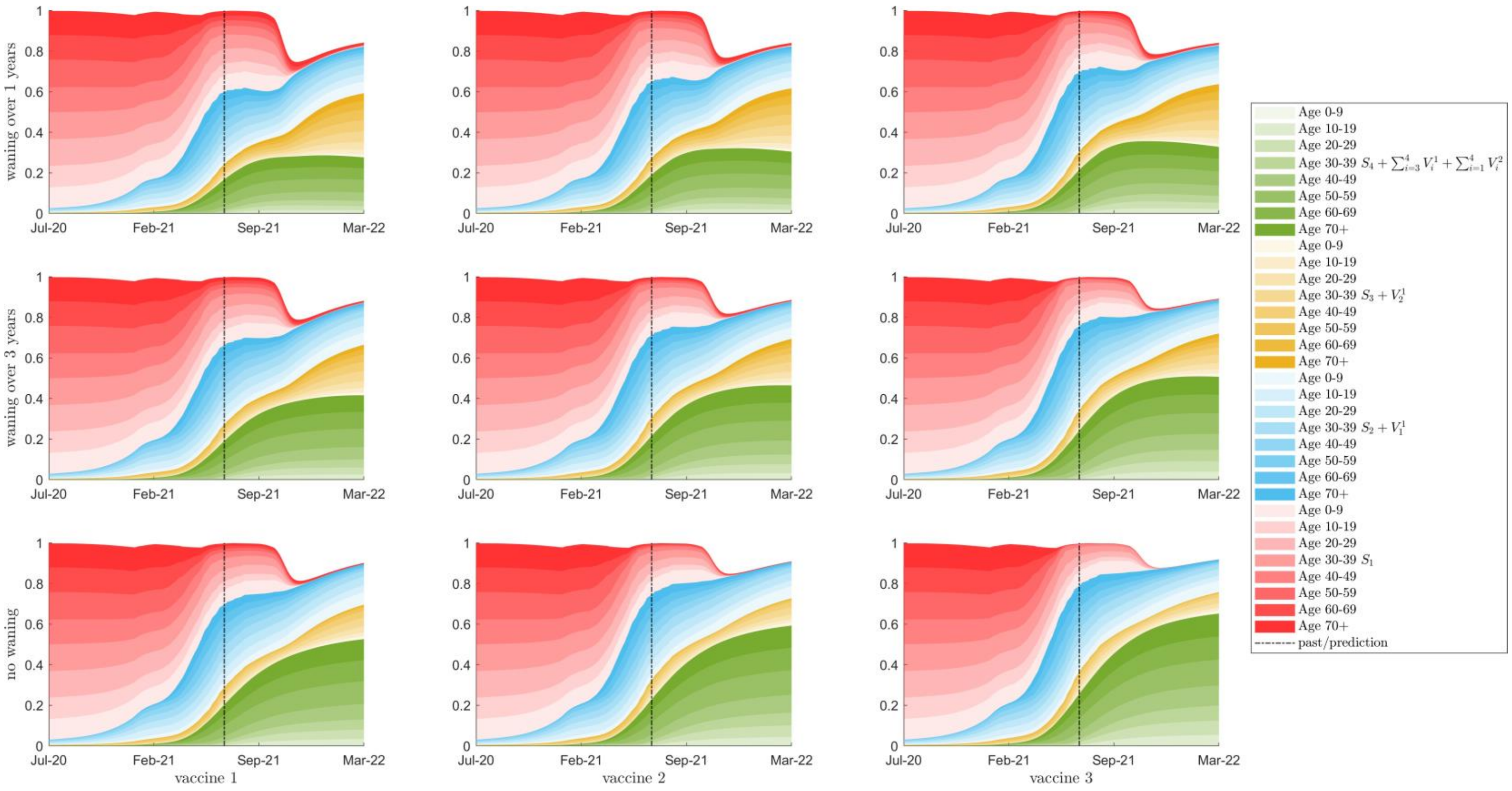

Figure S6: Distribution of Immunity, with a 38% relaxation of k starting in September 2021. Showing immune status of uninfected individuals as a percent of the total population for 10 year age classes, with colour intensity corresponding to age class. The colours represent the immunity of the population from green, the fully immune population, through yellow and blue to red, the fully susceptible population. The top row is waning of immunity by one year between consecutive classes; the middle row is waning of immunity of three years; the bottom row is no waning of immunity. Columns left to right represent vaccines 1 to 3, respectively. The vertical bar denotes the beginning of the prediction phase.
